# The HLA diversity of the Anthony Nolan register

**DOI:** 10.1111/tan.14127

**Published:** 2020-11-16

**Authors:** Gayle Leen, Jeremy E. Stein, James Robinson, Hazael Maldonado Torres, Steven G. E. Marsh

**Affiliations:** ^1^ Anthony Nolan Research Institute Royal Free Campus London UK; ^2^ UCL Cancer Institute Royal Free Campus London UK

**Keywords:** allele, diversity, haplotype, HLA, phenotype, United Kingdom

## Abstract

While the success of allogeneic stem cell transplantation depends on a high degree of HLA compatibility between donor and patient, finding a suitable donor remains challenging due to the hyperpolymorphic nature of HLA genes. We calculated high‐resolution allele, haplotype and phenotype frequencies for HLA‐A, ‐C, ‐B, ‐DRB1 and ‐DQB1 for 10 subpopulations of the Anthony Nolan (AN) register using an in‐house expectation‐maximisation (EM) algorithm run on mixed resolution HLA data, covering 676 155 individuals. Sample sizes range from 599 410 for British/Irish North West European (BINWE) individuals, the largest subpopulation in the United Kingdom to 1105 for the British Bangladeshi population. Calculation of genetic distance between the subpopulations based on haplotype frequencies shows three broad clusters, each following a major continental group: European, African and Asian. We further analysed the HLA haplotype and phenotype diversity of each subpopulation, and found that 35.52% of BINWE individuals ranging to 98.34% of Middle Eastern individuals on the register had a unique phenotype within their subpopulation. These analyses and the allele, haplotype and phenotype frequency data of the subpopulation on the AN register are a valuable resource in understanding the HLA diversity in the United Kingdom and can be used to improve the accuracy of match likelihoods and to inform future donor recruitment strategies.

## 1 INTRODUCTION

The Anthony Nolan (AN) register (https://www.anthonynolan.org), based in the United Kingdom (UK), was established in 1974 to facilitate unrelated haematopoietic stem cell transplantation (HSCT). As of February 2020, it has increased to more than 800 000 volunteer potential donors, representing over 1.27% of the UK population (based on a population size of 63 182 178^1^). Studies have shown that the success of allogeneic stem cell transplantation depends on a high degree of HLA compatibility between donor and patient, to avoid an adverse immune response that results in graft vs host disease,^2^, ^3^ with better outcomes for patients matched to donors at allelic resolution.^4^ Finding a suitably matched unrelated donor for a patient is challenging, due to the tremendous diversity of HLA genes.

The HLA genes, located within the human Major Histocompatibility Complex (MHC), encode proteins that play a critical role in immune system function.^5^ HLA molecules act as antigen presentation molecules by presenting self and non‐self‐peptides to T‐cells, which is the first step to initiating an adaptive immune response. HLA genes are some of the most polymorphic in the human genome, with the majority of the diversity found within the six classical HLA genes: HLA‐A, ‐C and ‐B (class I) and HLA‐DRB1, ‐DQB1 and ‐DPB1 (class II). As of February 2020, there are 26 326 known HLA alleles (19 115 class I and 7211 class II), with 7143 allelic variants reported for the most polymorphic locus, HLA‐B (https://www.ebi.ac.uk/ipd/imgt/hla/).^6^ An individual HLA allele can present a restricted repertoire of peptide motifs from either self or non‐self‐proteins to T‐cells. Differences between HLA alleles are predominantly due to differences in the peptide‐binding domain, encoded by exons 2 and 3 in HLA class I alleles, and exon 2 in HLA class II alleles. Analysis of nucleotide substitution patterns within the antigen recognition site (ARS) shows that the non‐synonymous (amino acid altering) substitution rate is significantly higher than the synonymous substitution rate in HLA class I^7^ and class II.^8^ This allelic variation, and subsequent variation in peptide binding repertoire, may lead to differences in immune response amongst individuals with different HLA phenotypes.

The hyperpolymorphism in HLA may have evolved due to pathogen‐driven balancing selection, in that the amino acid diversification of the peptide binding domain is a result of adaptation to a diverse range of pathogen species; increased diversity at HLA class I genes has been observed in populations living in geographical regions with high‐pathogen diversity.^9^ Levels of heterozygosity significantly higher than neutral expectations have been observed at HLA loci,^10^ suggesting that the high degree of polymorphism may be caused by overdominant selection (heterozygote advantage) where heterozygotes have higher fitness than homozygotes. Selective advantage against infectious disease is associated with HLA heterozygosity because individuals with a heterozygous genotype have a larger range of peptide motifs that can be presented to T‐cells, resulting in a more productive immune response to a diverse array of pathogens^11^ and it has been shown that maximal heterozygosity at the HLA class I loci slows progression to AIDS and AIDS‐related death.^12^ Additionally, divergent allele combinations may be advantageous,^13^, ^14^ since predictions of the total number of peptides bound by two alleles of a heterozygous genotype have found to be correlated with amino acid sequence divergence between the alleles.^15^ It has also been proposed that HLA polymorphism may be due to frequency dependent selection (rare allele advantage), where viruses adapt to HLA alleles that are common in the population, such that it is advantageous to have a rare allele to which the virus has not adapted as well.^16^, ^17^

The shape of the HLA allele frequency distribution of a population bears signatures of adaptation to its local environment and historical demographic events through genetic drift and the various methods of selection. By modelling genetic events, which lead to the creation of a novel allele through patterns of diversity in known alleles, it is possible to make predictions about the distribution of diversity. It has been predicted that the human population harbours around 8 to 9 million HLA class I variants, and that many alleles are rare and differ by point mutation from older, more common alleles.^18^ The HLA allele frequency spectrum has been proposed to consist of three frequency categories of alleles: common, purgatory and rare, with common alleles held in place by ongoing balancing selection, and a large population of rare alleles, which are subject to genetic drift and may only persist for a few generations. However, they may potentially assume epidemiologically meaningful roles following selective pressure by new and evolving pathogens.^19^

Within worldwide populations, there is extensive variation in HLA allele and haplotype frequencies due to geographical differences. Genetic distances based on HLA allele frequency distributions between outbred populations correlate with their geographical locations.^20^, ^21^ As well as varying in frequency between populations, some alleles and haplotypes are unique to an ethno‐linguistic group,^22^, ^23^ with regional differences observed in a single country.^24^ Studies have shown that the probability of finding an optimally matched unrelated donor varies with the ethnicity of the patient^25^, ^26^ due to different levels of diversity and representation on stem cell registries. Analysis of the NMDP registry shows that the likelihood of finding an available 8/8 HLA‐matched donor is 75% for people of European descent, but only 16‐19% for Americans of African descent from all ethnic backgrounds. Estimating the HLA diversity of a target population is essential in planning for the expansion of a stem cell registry and informing donor recruitment strategy, in order to maximise the likelihood of finding matches for patients.

HLA typing methodologies used for registry HLA typing have evolved over time, with earlier low‐resolution methods unable to determine the exact alleles present in an individual, resulting in allelic ambiguity and the reporting of a HLA typing string, while new typing methods offer allelic resolution.^27^ Ambiguous allele assignments result from either lack of sequence information at polymorphic positions that could disambiguate alleles, or inability to phase polymorphisms seen in an individual. Furthermore, typing for a locus may be missing due to change in registry typing strategy; for instance, donors recruited to the AN register have only been routinely typed for HLA‐C in the last 15 years, and HLA‐DQB1 in the last 5 years.

The range of typing methodologies and loci typed over the history of a register results in mixed resolution HLA types and missing data, which makes the calculation of allele and haplotype frequencies challenging. This has necessitated the tailoring of expectation‐maximisation (EM) algorithms^28^ to handle ambiguous typing data, where missing and ambiguous types are imputed based on the haplotype frequencies in the population. By imputing HLA types based on existing haplotypes in the population, and removing low‐probability haplotype pairs to achieve computational tractability, it is possible that there may be a bias towards common alleles and haplotypes, and an underestimation of HLA diversity. However, it has been shown that in registry datasets where a subset of donors was selected for additional typing on behalf of a patient, inclusion of all donors in the registry is less biassed than only including the high‐resolution typed subset, given that the overall mixed resolution typed cohort is unselected.^29^

In this article, we present allele and haplotype frequencies for 10 subpopulations of the AN register, calculated from mixed resolution data by using AN's in‐house EM‐based algorithm, Cactus.^30^, ^31^, ^32^ Due to the large sample size and the quality control procedures used in the HLA typing, we feel that it is a valuable resource for the HLA community.

## 2 METHODS AND MATERIALS

### 2.1 Study population and HLA typing

Data from the AN register was taken on 02/04/2019. Individuals were grouped by “Ethnicity”, a broad subpopulation based on the self‐declared ethnicity at the time of donor registration, with the number in each subpopulation, and fraction of each UK subpopulation in the register summarised in Table 1. Subpopulation sizes in the UK population were estimated using data from the 2011 census,^1^ using Tables 2011 Census: Ethnic group, local authorities in England and Wales (KS201EW), Ethnic group All people (KS201SC), and Ethnic Group (KS201NI). The size for the Jewish population in the United Kingdom was taken from KS209EW and KS209SCb. The ethnic groups as defined in the table KS201UK, which groups the ethnic groups across England, Wales, Scotland and Northern Ireland, were not used, since they do not map to all of the ethnic groups on the AN register; Supplementary Table S1 summarises the correspondence between the ethnicity on the AN register and the ethnic group used in this analysis.

**TABLE 1:** Summary of self‐defined ethnic groups on the Anthony Nolan (AN) register

Ethnicity	Size on AN Register	UK population size	Fraction of population in registry (%)
BINWE	599 410	52 424 292	1.143
Asian	37 505	3 940 189	0.952
India	10 597	1 451 862	0.730
Pakistan	3353	1 174 983	0.285
Bangladesh	1105	451 529	0.245
African	5761	1 021 611	0.564
African Caribbean	19 213	598 627	3.210
Jewish	9984	269 233	3.708
East Asian	4282	433 150	0.989
Middle Eastern	1449	239 966	0.604

Each individual is not necessarily assigned to a single group; for instance, the Asian population includes individuals from the Bangladesh, India and Pakistan groups, which are the three largest groups within the British Asian community. We did not include all data from the register; we only reported groups with a minimum of 1000 individuals. People from the BINWE (British/Irish/North West European) group make‐up the largest percentage of the UK population, and the largest proportion of the AN register. However, the proportion of the UK BINWE population on the AN register (1.14%) is not the highest of the subpopulations studied here; the proportion of the African Caribbean and Jewish populations on the register are 3.21% and 3.71%, respectively. The higher proportions are due to targeted recruitment drives such as the #Spit4Mum campaign in 2013 and the #BeingAfricanCaribbean campaign in 2015, focused on recruiting more Jewish and African Caribbean donors to the register respectively.

Each individual on the AN register has an HLA type which has been verified by in‐house clinical scientists. Across the whole register, different typing techniques have been used over time, such as serological, DNA‐based typing techniques (SSOP, SSP, SBT), and allelic resolution typing on the Pacific Biosciences RS II sequencing platform.^27^ In addition, the loci at which an individual is typed varies, reflecting changes in policy for tissue typing new donors over the lifetime of the register. Supplementary Table S2 summarises the sample size for each subpopulation and details the number of individuals typed at different combinations of HLA‐A, ‐C, ‐B, ‐DRB1 and ‐DQB1, where we only list individuals who have been typed at HLA‐A and ‐B as a minimum.

### 2.2 Data processing

HLA typing data for individuals on the AN register is mixed‐resolution data, ranging from serological (1 field) to allelic (4 field). Individuals who were missing typing at HLA‐A or HLA‐B were excluded from the subsequent analysis. In order to standardise each type, each typing string was transformed into its 2 field (ARS resolution) equivalent allele name, resulting in an ambiguous or unambiguous 2 field typing string. The latest optimisations of the AN software, Cactus,^30^, ^31^ was used to calculate haplotype frequencies using an EM approach.^28^, ^33^ Haplotype estimation was applied to sub‐haplotype blocks by incorporating loci in the following order: (HLA‐A ~ HLA‐B), ‐C, ‐DRB1 and ‐DQB1. After each imputation step, we retain possible haplotype pairs and probabilities for each donor up to 99.9999% cumulative probability. By using this blocks/imputation approach,^34^ our implementation reduced the number of possible haplotype pairs as haplotype blocks were extended, resulting in a computationally efficient algorithm. The software predicted a posterior distribution over possible ARS resolution diplotypes (HLA‐A, ‐B, ‐C, ‐DRB1, ‐DQB1) for each individual.

We calculated the allele frequencies for each locus, 5‐locus haplotype frequencies, and phenotype frequencies by summing over the diplotype distributions over all individuals. For haplotype and phenotype frequencies, we used a cutoff of 0.0001. We reported frequencies presented in the supplementary tables to 6 decimal places.

There are some limitations with the methodology used in this article. The accuracy of an individual's HLA diplotype or phenotype is dependent on the haplotype estimation procedure, the typing resolution, and whether imputation of missing loci is required. In this work, the algorithm resolves ambiguous allele or haplotype assignments by imputing alleles based on existing alleles in the population, and removes low‐probability haplotype pairs to achieve computational tractability. When estimating haplotype and allele frequencies, rare alleles and haplotypes may be underrepresented or missing.

### 2.3 Visualisation of results

**FIGURE 1:**
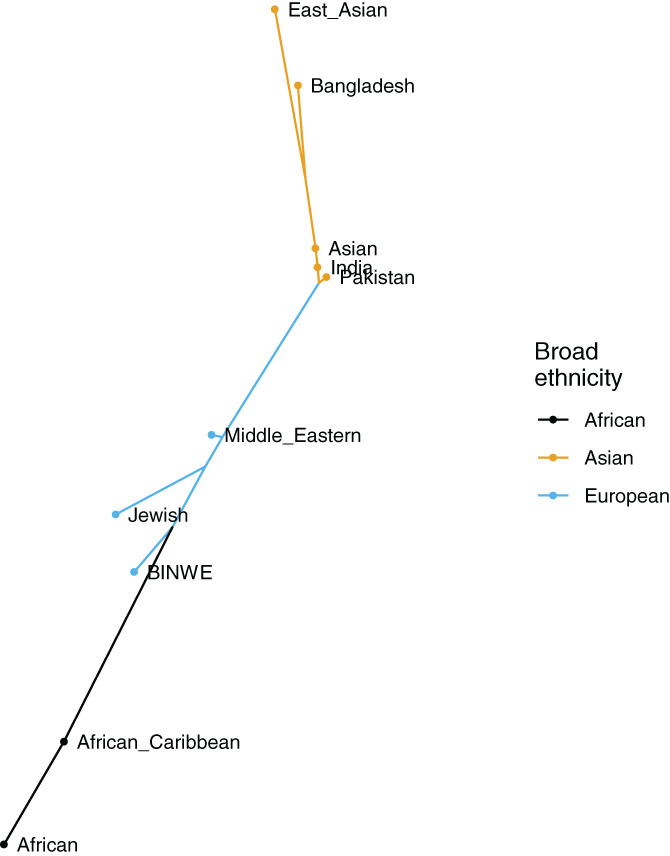
Nei's standard genetic distance calculated using haplotype frequencies for 10 subpopulations from the Anthony Nolan register

**FIGURE 2:**
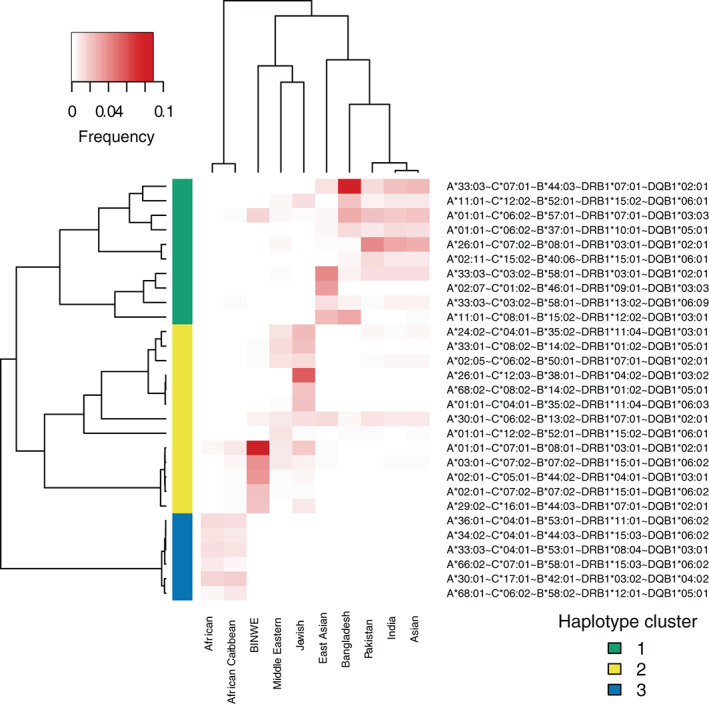
Visualisation of HLA haplotype sharing patterns in 10 subpopulations from the Anthony Nolan register

**FIGURE 3:**
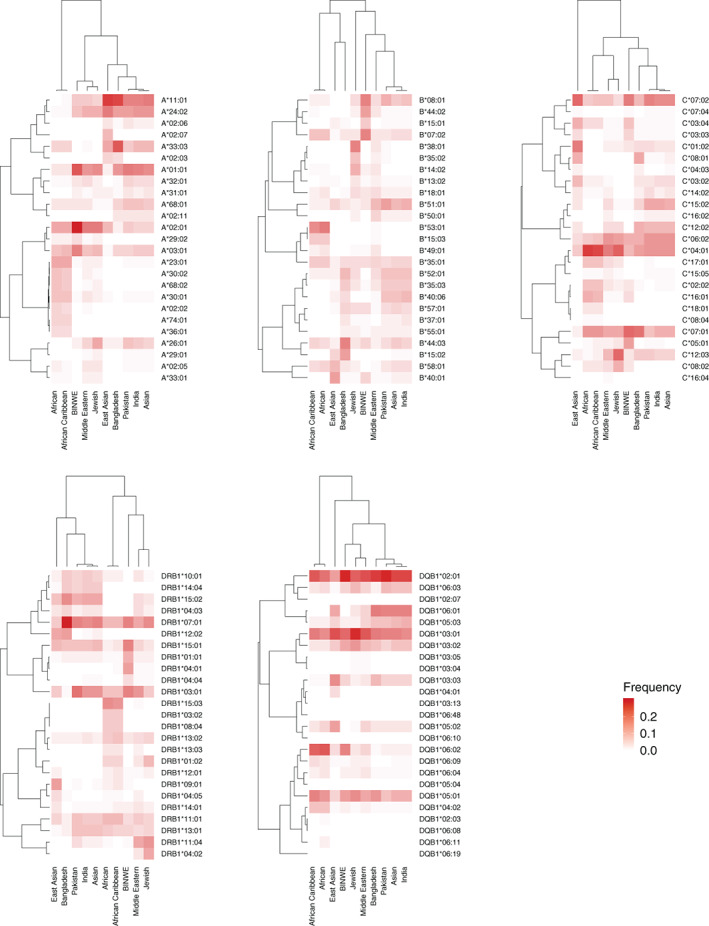
Visualisation of HLA‐A, ‐C, ‐B, ‐DRB1, ‐DQB1 allele sharing patterns in 10 subpopulation from the Anthony Nolan register

We used R^35^ with gplots^36^ to create the heatmaps in Figures 2 and 3, using Pearson correlation distance and UPGMA for the hierarchical clustering. We used R with APE^37^ for constructing the unrooted phylogenetic tree in Figure 1, using Nei's standard genetic distance^38^ calculated between 5‐locus haplotype frequency distribution for each subpopulation pair, and nearest neighbour joining. Any negative branch lengths were converted to 0. Ggtree^39^ was used to visualise the tree.

## 3 RESULTS

### 3.1 Allele and haplotype frequencies

HLA frequency data is presented for each subpopulation in the UK: BINWE, Asian and its three main subgroups India, Pakistan and Bangladesh, African, African Caribbean, Jewish, East Asian and Middle Eastern, based on the self‐identified ethnic group of donors at the time of registration. The complete frequency data (allele, haplotype and phenotype) for each subpopulation are available in the Supplementary Information as files freqs_{ethnicity}_results.xlsx.

Table 2 shows the top 10 HLA‐A ~ C ~ B ~ DRB1 ~ DQB1 haplotypes for each subpopulation, with corresponding frequencies across all groups. Only frequencies above 0.0001 are reported. The most frequently occurring haplotype observed within any population is A*01:01 ~ C*07:01 ~ B*08:01 ~ DRB1*03:01 ~ DQB1*02:01 in the BINWE population at a frequency of 8.41%, and is also common in the Jewish, African Caribbean, and Middle Eastern populations. The second most common haplotype in any population is A*33:03 ~ C*07:01 ~ B*44:03 ~ DRB1*07:01 ~ DQB1*02:01 occurs at a frequency of 7.90% in the Bangladesh population, and is common across the other Asian populations (Asian (2.26%), India (2.12%), Pakistan (1.34%)) and additionally in East Asian (1.02%), due to geographical proximity. The third most common haplotype in any population is A*26:01 ~ C*12:03 ~ B*38:01 ~ DRB1*04:02 ~ DQB1*03:02 in the Jewish population with a frequency of 5.28%. This haplotype is rare in other populations, though is seen in the Middle Eastern population at 0.14%.

**TABLE 2:** Top 10 haplotypes for each subpopulation and frequencies across all groups

Ethnicity	Rank	Haplotype	Frequency in population									
			BI	AS	IN	PA	BA	AF	CA	JE	EA	ME
BI	1	A*01:01 ~ C*07:01 ~ B*08:01 ~ DRB1*03:01 ~ DQB1*02:01	0.0841	0.0012	0.0005	0.0004		0.0033	0.0071	0.0178	0.0020	0.0068
	2	A*03:01 ~ C*07:02 ~ B*07:02 ~ DRB1*15:01 ~ DQB1*06:02	0.0332	0.0017	0.0019	0.0008		0.0003	0.0036	0.0063	0.0016	0.0066
	3	A*02:01 ~ C*05:01 ~ B*44:02 ~ DRB1*04:01 ~ DQB1*03:01	0.0297	0.0003	0.0003	0.0006		0.0010	0.0023	0.0032	0.0005	0.0031
	4	A*02:01 ~ C*07:02 ~ B*07:02 ~ DRB1*15:01 ~ DQB1*06:02	0.0207	0.0009	0.0010	0.0012			0.0024	0.0021		0.0022
	5	A*29:02 ~ C*16:01 ~ B*44:03 ~ DRB1*07:01 ~ DQB1*02:01	0.0201	0.0002		0.0006			0.0021	0.0081		0.0016
	6	A*01:01 ~ C*06:02 ~ B*57:01 ~ DRB1*07:01 ~ DQB1*03:03	0.0133	0.0195	0.0179	0.0211	0.0282	0.0005	0.0016	0.0022	0.0047	0.0039
	7	A*02:01 ~ C*07:01 ~ B*08:01 ~ DRB1*03:01 ~ DQB1*02:01	0.0101	0.0002	0.0002	0.0003	0.0005		0.0008	0.0088		0.0012
	8	A*03:01 ~ C*04:01 ~ B*35:01 ~ DRB1*01:01 ~ DQB1*05:01	0.0100	0.0037	0.0031	0.0028	0.0040		0.0011	0.0018	0.0012	
	9	A*02:01 ~ C*03:04 ~ B*15:01 ~ DRB1*04:01 ~ DQB1*03:02	0.0080					0.0002	0.0009	0.0012	0.0005	0.0017
	10	A*24:02 ~ C*07:02 ~ B*07:02 ~ DRB1*15:01 ~ DQB1*06:02	0.0079	0.0024	0.0024	0.0026	0.0016		0.0007	0.0009	0.0003	0.0018
AS	1	A*26:01 ~ C*07:02 ~ B*08:01 ~ DRB1*03:01 ~ DQB1*02:01		0.0259	0.0301	0.0388	0.0027		0.0001	0.0005	0.0022	0.0048
	2	A*33:03 ~ C*07:01 ~ B*44:03 ~ DRB1*07:01 ~ DQB1*02:01	0.0001	0.0226	0.0212	0.0134	0.0790		0.0010	0.0002	0.0102	
	3	A*01:01 ~ C*06:02 ~ B*57:01 ~ DRB1*07:01 ~ DQB1*03:03	0.0133	0.0195	0.0179	0.0211	0.0282	0.0005	0.0016	0.0022	0.0047	0.0039
	4	A*01:01 ~ C*06:02 ~ B*37:01 ~ DRB1*10:01 ~ DQB1*05:01	0.0016	0.0112	0.0133	0.0083	0.0136	0.0005	0.0007	0.0009	0.0044	0.0010
	5	A*33:03 ~ C*03:02 ~ B*58:01 ~ DRB1*03:01 ~ DQB1*02:01	0.0001	0.0112	0.0108	0.0117	0.0063	0.0008	0.0007		0.0396	0.0024
	6	A*02:11 ~ C*15:02 ~ B*40:06 ~ DRB1*15:01 ~ DQB1*06:01		0.0076	0.0083	0.0124	0.0045		0.0002		0.0011	0.0010
	7	A*30:01 ~ C*06:02 ~ B*13:02 ~ DRB1*07:01 ~ DQB1*02:01	0.0053	0.0073	0.0076	0.0095	0.0045	0.0007	0.0009	0.0115	0.0124	0.0076
	8	A*11:01 ~ C*12:02 ~ B*52:01 ~ DRB1*15:02 ~ DQB1*06:01	0.0006	0.0067	0.0077	0.0069	0.0204		0.0004	0.0115	0.0016	0.0042
	9	A*33:03 ~ C*03:02 ~ B*58:01 ~ DRB1*13:02 ~ DQB1*06:09	0.0004	0.0054	0.0064	0.0030	0.0059		0.0013		0.0115	0.0003
	10	A*01:01 ~ C*07:01 ~ B*15:17 ~ DRB1*13:02 ~ DQB1*06:04	0.0002	0.0047	0.0046	0.0060	0.0039		0.0001	0.0016		0.0033
IN	1	A*26:01 ~ C*07:02 ~ B*08:01 ~ DRB1*03:01 ~ DQB1*02:01		0.0259	0.0301	0.0388	0.0027		0.0001	0.0005	0.0022	0.0048
	2	A*33:03 ~ C*07:01 ~ B*44:03 ~ DRB1*07:01 ~ DQB1*02:01	0.0001	0.0226	0.0212	0.0134	0.0790		0.0010	0.0002	0.0102	
	3	A*01:01 ~ C*06:02 ~ B*57:01 ~ DRB1*07:01 ~ DQB1*03:03	0.0133	0.0195	0.0179	0.0211	0.0282	0.0005	0.0016	0.0022	0.0047	0.0039
	4	A*01:01 ~ C*06:02 ~ B*37:01 ~ DRB1*10:01 ~ DQB1*05:01	0.0016	0.0112	0.0133	0.0083	0.0136	0.0005	0.0007	0.0009	0.0044	0.0010
	5	A*33:03 ~ C*03:02 ~ B*58:01 ~ DRB1*03:01 ~ DQB1*02:01	0.0001	0.0112	0.0108	0.0117	0.0063	0.0008	0.0007		0.0396	0.0024
	6	A*02:11 ~ C*15:02 ~ B*40:06 ~ DRB1*15:01 ~ DQB1*06:01		0.0076	0.0083	0.0124	0.0045		0.0002		0.0011	0.0010
	7	A*11:01 ~ C*12:02 ~ B*52:01 ~ DRB1*15:02 ~ DQB1*06:01	0.0006	0.0067	0.0077	0.0069	0.0204		0.0004	0.0115	0.0016	0.0042
	8	A*30:01 ~ C*06:02 ~ B*13:02 ~ DRB1*07:01 ~ DQB1*02:01	0.0053	0.0073	0.0076	0.0095	0.0045	0.0007	0.0009	0.0115	0.0124	0.0076
	9	A*33:03 ~ C*03:02 ~ B*58:01 ~ DRB1*13:02 ~ DQB1*06:09	0.0004	0.0054	0.0064	0.0030	0.0059		0.0013		0.0115	0.0003
	10	A*01:01 ~ C*07:02 ~ B*08:01 ~ DRB1*03:01 ~ DQB1*02:01		0.0037	0.0048	0.0055			0.0001			0.0058
PA	1	A*26:01 ~ C*07:02 ~ B*08:01 ~ DRB1*03:01 ~ DQB1*02:01		0.0259	0.0301	0.0388	0.0027		0.0001	0.0005	0.0022	0.0048
	2	A*01:01 ~ C*06:02 ~ B*57:01 ~ DRB1*07:01 ~ DQB1*03:03	0.0133	0.0195	0.0179	0.0211	0.0282	0.0005	0.0016	0.0022	0.0047	0.0039
	3	A*33:03 ~ C*07:01 ~ B*44:03 ~ DRB1*07:01 ~ DQB1*02:01	0.0001	0.0226	0.0212	0.0134	0.0790		0.0010	0.0002	0.0102	
	4	A*02:11 ~ C*15:02 ~ B*40:06 ~ DRB1*15:01 ~ DQB1*06:01		0.0076	0.0083	0.0124	0.0045		0.0002		0.0011	0.0010
	5	A*33:03 ~ C*03:02 ~ B*58:01 ~ DRB1*03:01 ~ DQB1*02:01	0.0001	0.0112	0.0108	0.0117	0.0063	0.0008	0.0007		0.0396	0.0024
	6	A*30:01 ~ C*06:02 ~ B*13:02 ~ DRB1*07:01 ~ DQB1*02:01	0.0053	0.0073	0.0076	0.0095	0.0045	0.0007	0.0009	0.0115	0.0124	0.0076
	7	A*32:01 ~ C*07:02 ~ B*08:01 ~ DRB1*03:01 ~ DQB1*02:01		0.0035	0.0024	0.0089						
	8	A*01:01 ~ C*06:02 ~ B*37:01 ~ DRB1*10:01 ~ DQB1*05:01	0.0016	0.0112	0.0133	0.0083	0.0136	0.0005	0.0007	0.0009	0.0044	0.0010
	9	A*11:01 ~ C*12:02 ~ B*52:01 ~ DRB1*15:02 ~ DQB1*06:01	0.0006	0.0067	0.0077	0.0069	0.0204		0.0004	0.0115	0.0016	0.0042
	10	A*01:01 ~ C*07:01 ~ B*15:17 ~ DRB1*13:02 ~ DQB1*06:04	0.0002	0.0047	0.0046	0.0060	0.0039		0.0001	0.0016		0.0033
BA	1	A*33:03 ~ C*07:01 ~ B*44:03 ~ DRB1*07:01 ~ DQB1*02:01	0.0001	0.0226	0.0212	0.0134	0.0790		0.0010	0.0002	0.0102	
	2	A*11:01 ~ C*08:01 ~ B*15:02 ~ DRB1*12:02 ~ DQB1*03:01		0.0031	0.0008	0.0013	0.0308	0.0001			0.0221	
	3	A*01:01 ~ C*06:02 ~ B*57:01 ~ DRB1*07:01 ~ DQB1*03:03	0.0133	0.0195	0.0179	0.0211	0.0282	0.0005	0.0016	0.0022	0.0047	0.0039
	4	A*11:01 ~ C*12:02 ~ B*52:01 ~ DRB1*15:02 ~ DQB1*06:01	0.0006	0.0067	0.0077	0.0069	0.0204		0.0004	0.0115	0.0016	0.0042
	5	A*01:01 ~ C*06:02 ~ B*37:01 ~ DRB1*10:01 ~ DQB1*05:01	0.0016	0.0112	0.0133	0.0083	0.0136	0.0005	0.0007	0.0009	0.0044	0.0010
	6	A*11:01 ~ C*08:01 ~ B*15:02 ~ DRB1*15:01 ~ DQB1*06:01		0.0018	0.0011	0.0028	0.0135				0.0062	
	7	A*02:03 ~ C*08:01 ~ B*15:02 ~ DRB1*15:02 ~ DQB1*05:01		0.0008	0.0006		0.0090				0.0002	
	8	A*24:02 ~ C*07:01 ~ B*44:03 ~ DRB1*07:01 ~ DQB1*02:01		0.0019	0.0017	0.0004	0.0082				0.0003	
	9	A*11:01 ~ C*07:01 ~ B*44:03 ~ DRB1*07:01 ~ DQB1*02:01		0.0041	0.0040	0.0039	0.0073				0.0015	
	10	A*24:02 ~ C*07:02 ~ B*07:02 ~ DRB1*07:01 ~ DQB1*02:01	0.0004	0.0017	0.0013	0.0015	0.0064		0.0002		0.0013	
AF	1	A*30:01 ~ C*17:01 ~ B*42:01 ~ DRB1*03:02 ~ DQB1*04:02		0.0002		0.0006		0.0130	0.0145		0.0001	0.0014
	2	A*36:01 ~ C*04:01 ~ B*53:01 ~ DRB1*11:01 ~ DQB1*06:02						0.0111	0.0102			
	3	A*33:03 ~ C*04:01 ~ B*53:01 ~ DRB1*08:04 ~ DQB1*03:01						0.0103	0.0086			
	4	A*34:02 ~ C*04:01 ~ B*44:03 ~ DRB1*15:03 ~ DQB1*06:02						0.0086	0.0071			
	5	A*66:02 ~ C*07:01 ~ B*58:01 ~ DRB1*15:03 ~ DQB1*06:02						0.0066	0.0044			
	6	A*68:01 ~ C*06:02 ~ B*58:02 ~ DRB1*12:01 ~ DQB1*05:01						0.0047	0.0067			
	7	A*68:02 ~ C*03:04 ~ B*15:10 ~ DRB1*03:01 ~ DQB1*02:01		0.0001				0.0040	0.0044			
	8	A*36:01 ~ C*04:01 ~ B*53:01 ~ DRB1*15:03 ~ DQB1*06:02						0.0037	0.0019			
	9	A*30:01 ~ C*17:01 ~ B*42:01 ~ DRB1*08:04 ~ DQB1*03:01						0.0037	0.0037			0.0003
	10	A*23:01 ~ C*07:02 ~ B*07:02 ~ DRB1*09:01 ~ DQB1*02:01						0.0036	0.0040			
CA	1	A*30:01 ~ C*17:01 ~ B*42:01 ~ DRB1*03:02 ~ DQB1*04:02		0.0002		0.0006		0.0130	0.0145		0.0001	0.0014
	2	A*36:01 ~ C*04:01 ~ B*53:01 ~ DRB1*11:01 ~ DQB1*06:02						0.0111	0.0102			
	3	A*33:03 ~ C*04:01 ~ B*53:01 ~ DRB1*08:04 ~ DQB1*03:01						0.0103	0.0086			
	4	A*34:02 ~ C*04:01 ~ B*44:03 ~ DRB1*15:03 ~ DQB1*06:02						0.0086	0.0071			
	5	A*01:01 ~ C*07:01 ~ B*08:01 ~ DRB1*03:01 ~ DQB1*02:01	0.0841	0.0012	0.0005	0.0004		0.0033	0.0071	0.0178	0.0020	0.0068
	6	A*68:01 ~ C*06:02 ~ B*58:02 ~ DRB1*12:01 ~ DQB1*05:01						0.0047	0.0067			
	7	A*68:02 ~ C*03:04 ~ B*15:10 ~ DRB1*03:01 ~ DQB1*02:01		0.0001				0.0040	0.0044			
	8	A*66:02 ~ C*07:01 ~ B*58:01 ~ DRB1*15:03 ~ DQB1*06:02						0.0066	0.0044			
	9	A*23:01 ~ C*07:02 ~ B*07:02 ~ DRB1*09:01 ~ DQB1*02:01						0.0036	0.0040			
	10	A*30:01 ~ C*17:01 ~ B*42:01 ~ DRB1*08:04 ~ DQB1*03:01						0.0037	0.0037			0.0003
JE	1	A*26:01 ~ C*12:03 ~ B*38:01 ~ DRB1*04:02 ~ DQB1*03:02	0.0006	0.0001						0.0528		0.0014
	2	A*33:01 ~ C*08:02 ~ B*14:02 ~ DRB1*01:02 ~ DQB1*05:01	0.0022	0.0014	0.0016	0.0016		0.0006	0.0008	0.0222	0.0006	0.0124
	3	A*24:02 ~ C*04:01 ~ B*35:02 ~ DRB1*11:04 ~ DQB1*03:01	0.0010	0.0032	0.0034	0.0049			0.0003	0.0212		0.0087
	4	A*01:01 ~ C*04:01 ~ B*35:02 ~ DRB1*11:04 ~ DQB1*06:03	0.0002							0.0199	0.0002	0.0017
	5	A*68:02 ~ C*08:02 ~ B*14:02 ~ DRB1*01:02 ~ DQB1*05:01	0.0003							0.0193		
	6	A*01:01 ~ C*07:01 ~ B*08:01 ~ DRB1*03:01 ~ DQB1*02:01	0.0841	0.0012	0.0005	0.0004		0.0033	0.0071	0.0178	0.0020	0.0068
	7	A*01:01 ~ C*06:02 ~ B*57:01 ~ DRB1*13:05 ~ DQB1*03:01	0.0002							0.0177		
	8	A*02:01 ~ C*04:01 ~ B*35:03 ~ DRB1*12:01 ~ DQB1*03:01	0.0003							0.0177		
	9	A*03:01 ~ C*12:03 ~ B*38:01 ~ DRB1*13:01 ~ DQB1*06:03	0.0004	0.0003	0.0003					0.0163		0.0007
	10	A*02:05 ~ C*06:02 ~ B*50:01 ~ DRB1*07:01 ~ DQB1*02:01	0.0029	0.0044	0.0046	0.0024	0.0005		0.0002	0.0120	0.0016	0.0088
EA	1	A*33:03 ~ C*03:02 ~ B*58:01 ~ DRB1*03:01 ~ DQB1*02:01	0.0001	0.0112	0.0108	0.0117	0.0063	0.0008	0.0007		0.0396	0.0024
	2	A*02:07 ~ C*01:02 ~ B*46:01 ~ DRB1*09:01 ~ DQB1*03:03		0.0006							0.0303	
	3	A*11:01 ~ C*08:01 ~ B*15:02 ~ DRB1*12:02 ~ DQB1*03:01		0.0031	0.0008	0.0013	0.0308	0.0001			0.0221	
	4	A*30:01 ~ C*06:02 ~ B*13:02 ~ DRB1*07:01 ~ DQB1*02:01	0.0053	0.0073	0.0076	0.0095	0.0045	0.0007	0.0009	0.0115	0.0124	0.0076
	5	A*33:03 ~ C*03:02 ~ B*58:01 ~ DRB1*13:02 ~ DQB1*06:09	0.0004	0.0054	0.0064	0.0030	0.0059		0.0013		0.0115	0.0003
	6	A*33:03 ~ C*07:01 ~ B*44:03 ~ DRB1*07:01 ~ DQB1*02:01	0.0001	0.0226	0.0212	0.0134	0.0790		0.0010	0.0002	0.0102	
	7	A*11:01 ~ C*01:02 ~ B*46:01 ~ DRB1*09:01 ~ DQB1*03:03		0.0002							0.0083	
	8	A*02:03 ~ C*07:02 ~ B*38:02 ~ DRB1*16:02 ~ DQB1*05:02		0.0005	0.0002	0.0003					0.0072	
	9	A*11:01 ~ C*07:02 ~ B*40:01 ~ DRB1*09:01 ~ DQB1*03:03		0.0001							0.0067	
	10	A*11:01 ~ C*03:04 ~ B*13:01 ~ DRB1*15:01 ~ DQB1*06:01		0.0002							0.0067	
ME	1	A*33:01 ~ C*08:02 ~ B*14:02 ~ DRB1*01:02 ~ DQB1*05:01	0.0022	0.0014	0.0016	0.0016		0.0006	0.0008	0.0222	0.0006	0.0124
	2	A*01:01 ~ C*12:02 ~ B*52:01 ~ DRB1*15:02 ~ DQB1*06:01	0.0017	0.0023	0.0016	0.0036	0.0037		0.0004	0.0034	0.0011	0.0089
	3	A*02:05 ~ C*06:02 ~ B*50:01 ~ DRB1*07:01 ~ DQB1*02:01	0.0029	0.0044	0.0046	0.0024	0.0005		0.0002	0.0120	0.0016	0.0088
	4	A*24:02 ~ C*04:01 ~ B*35:02 ~ DRB1*11:04 ~ DQB1*03:01	0.0010	0.0032	0.0034	0.0049			0.0003	0.0212		0.0087
	5	A*30:01 ~ C*06:02 ~ B*13:02 ~ DRB1*07:01 ~ DQB1*02:01	0.0053	0.0073	0.0076	0.0095	0.0045	0.0007	0.0009	0.0115	0.0124	0.0076
	6	A*01:01 ~ C*07:01 ~ B*08:01 ~ DRB1*03:01 ~ DQB1*02:01	0.0841	0.0012	0.0005	0.0004		0.0033	0.0071	0.0178	0.0020	0.0068
	7	A*03:01 ~ C*07:02 ~ B*07:02 ~ DRB1*15:01 ~ DQB1*06:02	0.0332	0.0017	0.0019	0.0008		0.0003	0.0036	0.0063	0.0016	0.0066
	8	A*01:01 ~ C*07:02 ~ B*08:01 ~ DRB1*03:01 ~ DQB1*02:01		0.0037	0.0048	0.0055			0.0001			0.0058
	9	A*01:01 ~ C*17:01 ~ B*41:01 ~ DRB1*07:01 ~ DQB1*03:03	0.0003			0.0004			0.0001	0.0003	0.0004	0.0056
	10	A*02:01 ~ C*12:02 ~ B*52:01 ~ DRB1*15:02 ~ DQB1*06:01	0.0005	0.0020	0.0021	0.0031		0.0007	0.0003	0.0086		0.0050

Abbreviations: AF, African; AS, Asian; BA, Bangladesh; BI, BINWE; CA, African Caribbean; EA, East Asian; IN, India; JE, Jewish; ME, Middle Eastern; PA, Pakistan.

We explored the relationships between the subpopulations in the UK based on haplotype frequency distributions. This is visualised in Figure 1, which shows Nei's standard genetic distance calculated for each pair of subpopulations using haplotype frequencies (see Supplementary Table S3), represented as a phylogenetic tree obtained by the nearest neighbour approach. We found that the subpopulations can be clustered into three main groups: African/African Caribbean, BINWE/Jewish/Middle Eastern, and East Asian/Bangladesh/Pakistan/India/Asian, which we have indicated with three coloured subtrees, each following a major continental group: African, European and Asian. Within the Asian subtree, the shorter branches between the Asian, Indian and Pakistan populations suggest that these populations were similar to each other. This is plausible because immigrants from the Punjab region (comprising areas of eastern Pakistan and northern India) are one of the largest subpopulations amongst British Asians,^40^ and the Asian population category contains all the South Asian populations (see Supplementary Table S1), with Indian and Pakistani populations as the largest groups.

We visualised HLA‐A ~ C ~ B ~ DRB1 ~ DQB1 haplotype sharing patterns between the 10 subpopulations in Figure 2. We took the top 5 haplotypes for each group, removing any duplicates, to give a set of 29 common haplotypes. We clustered the haplotypes based on similarities between the frequency profiles of these haplotypes across the subpopulations, and clustered the subpopulations based on the frequency profiles across the common haplotype set (Pearson correlation distance used for both clusterings). As expected, the subpopulations were clustered into the three main groups (African, Asian and European) as found before, each with a set of haplotypes that occurs frequently in at least one of the populations in the group (Haplotype clusters 1‐3). The African group was the most different to the other groups, with a cluster of haplotypes (Haplotype cluster 3) that occurred frequently in this group but were rare in other populations. Within the Asian group, haplotype cluster 2 could be split into two, where one cluster had haplotypes more commonly associated with East Asia such as A*02:07 ~ C*01:02 ~ B*46:01 ~ DRB1*09:01 ~ DQB1*03:03 and A*33:03 ~ C*03:02 ~ B*58:01 ~ DRB1*03:01 ~ DQB1*02:01, while the other had haplotypes more commonly associated with the South Asian countries. The Bangladesh group had frequently occurring haplotypes associated with both subclusters such as A*01:01 ~ C*06:02 ~ B*57:01 ~ DRB1*07:01 ~ DQB1*03:03 (South Asian) and A*11:01 ~ B*15:02 ~ C*08:01 ~ DRB1*12:02 ~ DQB1*03:01 (East Asian).

We counted the number of alleles at each locus for each subpopulation, and the results are presented in Table 3. The HLA‐B locus had the highest allelic diversity, with 59 distinct alleles observed in British Bangladeshis, ranging to 407 alleles in the BINWE population. HLA‐DQB1 had the lowest allelic diversity of the loci studied. Overall, the diversity of the entire population (combined into Ethnicity: All in Table 3) only represents a small fraction of worldwide HLA diversity, described in the IPD‐IMGT/HLA Database (version 3.39.0). At the protein level, 356/3720 (9.57%) of known alleles are observed for HLA‐A, 303/3470 (8.73%) for C, 497/4604 (10.79%) for B, 281/1889 (14.88%) for DRB1 and 105/1194 (8.79%) for DQB1. We visualised allele sharing patterns in the 10 subpopulations in Figure 3. We took the top 20 alleles across all subpopulations and clustered the alleles based on similarities between the frequency profiles of alleles across the ethnic groups, and clustered the subpopulations based on the frequency profiles across the common allele set.

**TABLE 3:** Number of alleles observed in 10 subpopulations on the AN register

Ethnicity	Number of alleles observed				
	A	C	B	DRB1	DQB1
BINWE	277	243	407	230	84
Asian	97	82	166	114	37
India	65	57	104	78	27
Pakistan	52	39	82	61	19
Bangladesh	29	33	59	43	16
African	65	35	99	52	19
African Caribbean	105	67	155	84	28
Jewish	44	29	82	45	18
East Asian	54	44	111	64	23
Middle Eastern	46	32	79	51	17
All	356	303	497	281	105

### 3.2 Haplotype and phenotype diversity

Haplotypes that only occur once in a population that is, unique to one individual, known as singletons, can be indicative of the population's diversity. We estimated an unambiguous diplotype (pair of haplotypes) for each individual by taking the diplotype with the maximum probability. The proportions of the unique haplotypes in each subpopulation which were singletons are shown in Table 4. In the BINWE population, there were 29 028 unique haplotypes, of which 12 773 were singletons (44.00%, only occur once). The BINWE population had the lowest proportion of singletons, and the Middle Eastern population had the highest proportion of singletons (73.33%). These proportions are affected by the sample size of the subpopulations. To compare the diversity of the subpopulations with equal sample size, we sampled 1000 individuals from each subpopulation and calculated the proportion of the haplotypes which were singletons. We also calculated 95% confidence intervals for this statistic for 10 000 random samples from each population. In Table 5 we report the median proportion of haplotypes which are singletons and the 95% confidence interval for each subpopulation. With sample size equal in each subpopulation, the Jewish and Bangladeshi populations have the lowest haplotype diversity, with the highest diversity seen in the African Caribbean and Middle Eastern populations.

**TABLE 4:** Summary of haplotype and phenotype diversity on the AN register

Ethnicity	Number of unique haplotypes	Haplotypes that are singletons (%)	Number of unique phenotypes	Phenotypes that are singletons (%)	Fraction of population that are phenotype singletons (%)
BINWE	29 028	44.002	273 195	77.929	35.518
Asian	10 876	50.432	32 906	91.530	80.307
India	5184	56.694	9889	94.539	88.223
Pakistan	2371	61.620	3198	96.185	91.739
Bangladesh	961	66.701	1035	95.556	89.502
African	3996	56.707	5635	97.888	95.747
African Caribbean	8849	52.808	18 270	96.092	91.376
Jewish	2510	48.367	7612	85.234	64.984
East Asian	3125	66.016	4074	96.760	92.060
Middle Eastern	1680	73.333	1437	99.165	98.344

**TABLE 5:** Comparison of haplotype diversity between subpopulations on the AN register. We compared the proportion of haplotypes that are singletons for each subpopulation on the AN register, for 1000 individuals, sampled from the full subpopulation size. We report 95% confidence intervals over 10 000 random samples

Ethnicity	Haplotypes that are singletons (%)	95% CI
BINWE	72.033	(69.444, 74.437)
Asian	74.735	(72.579, 76.831)
India	73.521	(71.390, 75.645)
Pakistan	71.065	(68.852, 73.164)
Bangladesh	67.937	(66.704, 69.214)
African	75.079	(73.077, 76.977)
African Caribbean	77.490	(75.525, 79.382)
Jewish	64.222	(61.194, 67.066)
East Asian	76.418	(74.406, 78.412)
Middle Eastern	76.730	(75.240, 78.159)

We estimated the most likely phenotype for each individual on the AN register, using the haplotype frequencies. Similarly to the haplotype diversity analysis, we calculated the proportions of unique phenotypes in each subpopulation, which are singletons, with the results in Table 4. In addition, we calculated the proportion of each population, which have a unique phenotype. Again, we found that phenotype diversity in these populations followed that of haplotype diversity; 35.52% of the BINWE individuals on the registry had a unique phenotype. The Middle Eastern population had the highest proportion of singletons (98.34%). As before, to compare the diversity of the subpopulations with equal sample size, we sampled 1000 individuals from each subpopulation and calculated the proportion of the 1000 individuals with a unique phenotype. We also calculated 95% confidence intervals for this statistic for 10 000 random samples from each population and the results are shown in Table 6. In this analysis, the BINWE, Bangladeshi and Jewish populations have the lowest phenotype diversity, and the African Caribbean and African population have the highest. The proportion of singletons in a population affects the likelihood of finding a phenotype match in the registry, and therefore it is important to estimate the amount of diversity in a population.

**TABLE 6:** Comparison of phenotype diversity between subpopulations on the AN register. We compared the proportion of the population that are phenotype singletons for each subpopulation on the AN register for 1000 individuals, sampled from the full subpopulation size. We report 95% confidence intervals over 10 000 random samples

Ethnicity	Fraction of population that are phenotype singletons (%)	95% CI
BINWE	90.300	(87.800, 92.500)
Asian	98.500	(97.300, 99.400)
India	98.300	(97.000, 99.400)
Pakistan	96.900	(95.400, 98.200)
Bangladesh	90.100	(89.200, 91.100)
African	99.200	(98.400, 99.800)
African Caribbean	99.300	(98.400, 100.000)
Jewish	89.900	(87.300, 92.300)
East Asian	96.800	(95.400, 98.100)
Middle Eastern	98.800	(98.200, 99.600)

## 4 DISCUSSION

The AN register aims to provide stem cell donors for patients in the United Kingdom. People of various ethnicities reside in the United Kingdom, reflecting historical mass migration events of the twentieth century such as post‐World War II Commonwealth immigration. Given the ethnically diverse make‐up and the subsequent HLA diversity of the UK population, donor recruitment strategies may have to be population‐specific in order to optimally fulfil the demands of UK patients requiring a stem cell transplant. Analysis of the HLA diversity of the different subpopulations on the AN register is an important step in assessing if the current registry has sufficient sampling depth to represent each subpopulation. We calculated 5‐locus HLA‐A ~ C ~ B ~ DRB1 ~ DQB1 haplotype frequencies for the main subpopulations on the AN register, and presented these frequencies, along with allele and phenotype frequencies, forming a valuable reference set.

At the protein level, we found that only a small fraction of worldwide HLA allelic diversity is represented by the entire AN register, ranging from 8.73% of known HLA‐C alleles to 14.88% of known HLA‐DRB1 alleles; a large fraction of known alleles are not currently seen amongst the subpopulations on the register. However, we found that there is great diversity when studying haplotypes and phenotypes of the register; for the largest, and one of the least diverse subpopulations, the BINWE population, 35.52% of individuals have a unique phenotype. For a subpopulation with high‐HLA diversity, the likelihood of finding a phenotype match for individuals of that ethnicity will be small. However, when making inferences about the diversity of the AN register, we acknowledge that the results are from the processing of mixed resolution HLA data, where ambiguous and missing data have been imputed based on the rest of the population. It is possible that this representation could bias the estimates of allele, haplotype and phenotype frequencies towards the most common and underestimate the levels of diversity in the population.

In this work, the HLA type for each individual was estimated for HLA‐A, ‐C, ‐B, ‐DRB1 and ‐DQB1, to 2‐field (ARS) resolution, such that finding a matching type for an individual is equivalent to at least a 10/10 match at 2‐field resolution. HLA‐DPB1 was not included in the analysis due to its historic absence in typings on the register, though matching at HLA‐DPB1^3^ and permissive HLA‐DPB1 mismatching^41^ have been shown to be beneficial. However, finding a 10/10 donor with matched or permissively matched HLA‐DPB1 can be challenging due to a recombination hotspot between HLA‐DQB1 and DPB1. In addition, matching outside the ARS by moving to an allelic‐level resolution typing strategy can significantly improve outcomes for recipients of a stem cell transplant.^4^ Employing stricter matching criteria by including extra loci such as HLA‐DPB1 in this analysis, or increasing the typing resolution, would increase the haplotype and phenotype diversity, reducing the probability of finding a phenotype match on the register. However, examination of the haplotype frequency distributions showed shared haplotypes between different groups (Table 2), so it is possible that matches could be found in individuals from other ethnic backgrounds on the register. In this article, we have focused on characterising the HLA diversity for each subpopulation of the AN register. However, it is not only HLA diversity within a subpopulation that impacts phenotype match likelihoods; genetic distance from the other subpopulations represented on the register influences the probability of finding a match. Future work will focus on quantifying the overlap between different subpopulations and determining phenotype match likelihoods within and between populations on the register. In addition, since in practise many donors are found through searching international registries, particularly if the recipient's subpopulation is underrepresented on the AN register, an analysis of match likelihoods between different subpopulations on different registries could be beneficial.

Characterising the haplotype frequency distributions for each of the main ethnic groups in the United Kingdom enables us to analyse individuals of mixed origins in the United Kingdom in future work as an admixture of groups. At 1 250 230 individuals^1^ (1.98% of the UK population), individuals of mixed origins are the fastest growing ethnic minority group in the United Kingdom, evidence that the UK is becoming more ethnically diverse. By quantifying the different levels of HLA diversity in the different subpopulations in the United Kingdom population, we have taken a valuable first step to quantifying and identifying coverage gaps in the AN register, providing information which may influence future donor recruitment strategies in response to the UK's changing HLA profile.

## CONFLICT OF INTEREST

The authors have declared no conflicting interests.

## AUTHOR CONTRIBUTIONS

James Robinson and Steven G. E. Marsh conceived the study. Hazael Maldonado Torres, Jeremy E. Stein and Gayle Leen wrote the software for processing and estimating haplotype frequencies from the registry data. Gayle Leen analysed the data and wrote the manuscript. All authors reviewed the manuscript.

## Supporting information


**Table S1** Supporting Information

**Table S2** Supporting Information

**Table S3** Supporting Information

Supporting Information

## Data Availability

Research data for this project is not shared. Summary data representing the frequencies of alleles, haplotypes and phenotypes generated as part of this study are available in the supplementary material of this article.
