# Supplementary material for: The HLA diversity of the Anthony Nolan register
Source: HLA. 2020 Nov 16;97(1):15–29. doi: 10.1111/tan.14127 (PMC7756289; doi:10.1111/tan.14127)
Supplement: Supplementary file 1 — Table S1 Supporting Information [file TAN-97-15-s001.pdf]

S 1: Mapping between broad ethnic group used in this analysis, to ethnic categories from UK 2011 Census data, England and Wales (Table KS201EW), Scotland (Table KS201SC), Northern Ireland (Table KS201NI) and ethnic groups on the AN register

Ethnic group	Ethnicities on AN register	England/Wales category	Scotland category	Northern Ireland category
Asian	Asian, Pakistan, India, Bangladesh, Other South Asian, Southern Asian	Asian/Asian British: Other Asian, Asian/Asian British: Bangladeshi, Asian/Asian British: Indian, Asian/Asian British: Pakistani	Asian, Asian Scottish or Asian British: Other Asian, Asian, Asian Scottish or Asian British: Bangladeshi, Bangladeshi Scottish or Bangladeshi British, Asian, Asian Scottish or Asian British: Indian, Indian Scottish or Indian British, Asian, Asian Scottish or Asian British: Pakistani, Pakistani Scottish or Pakistani British	Other Asian, Bangladeshi, Indian, Pakistani
BINWE	British/Irish, N European, Other Northern European	White: English/Welsh/Scottish /Northern Irish/British, White: Irish, White: Gypsy or Irish Traveller	White: Scottish, White: Other British, White: Irish, White: Gypsy/Traveller	White, Irish Traveller
Bangladesh	Bangladesh	Asian/Asian British: Bangladeshi	Asian, Asian Scottish or Asian British: Bangladeshi, Bangladeshi Scottish or Bangladeshi British	Bangladeshi
African	African	Black/African/Caribbean/Bla British: African	African	African
Caribbean	African-Caribbean	Black/African/Caribbean/Bla British: Caribbean	Caribbean or Black: Caribbean, Caribbean Scottish or Caribbean British	Caribbean
India	India	Asian/Asian British: Indian	Asian, Asian Scottish or Asian British: Indian, Indian Scottish or Indian British	Indian
Middle Eastern	Middle Eastern	Other ethnic group: Arab	Other ethnic groups: Arab, Arab Scottish or Arab British	
East Asian	Oriental, East Asian, Southeast Asia	Asian/Asian British: Chinese	Asian, Asian Scottish or Asian British: Chinese, Chinese Scottish or Chinese British	Chinese
Pakistan	Pakistan	Asian/Asian British: Pakistani	Asian, Asian Scottish or Asian British: Pakistani, Pakistani Scottish or Pakistani British	Pakistani
