# Supplementary material for: The HLA diversity of the Anthony Nolan register
Source: HLA. 2020 Nov 16;97(1):15–29. doi: 10.1111/tan.14127 (PMC7756289; doi:10.1111/tan.14127)
Supplement: Supplementary file 2 — Table S2 Supporting Information [file TAN-97-15-s002.pdf]

S 2: Breakdown of HLA typing data by ethnicity

Ethnicity	Typed at					Number of individuals	
	A	C	B	DRB1	DQB1	Count	Fraction (%)
BINWE	X	X	X	X	X	299703	50.00
	X	X	X	X		138725	23.14
	X	X	X		X	264	0.04
	X	X	X			194	0.03
	X		X	X	X	17968	3.00
	X		X	X		120618	20.12
	X		X		X	9	0.00
	X		X			21929	3.66
Asian	X	X	X	X	X	22451	59.86
	X	X	X	X		8603	22.94
	X	X	X		X	46	0.12
	X	X	X			24	0.06
	X		X	X	X	328	0.87
	X		X	X		5582	14.88
	X		X		X	7	0.02
	X		X			464	1.24
India	X	X	X	X	X	10352	97.69
	X	X	X	X		202	1.91
	X	X	X		X	16	0.15
	X	X	X			3	0.03
	X		X	X	X	8	0.08
	X		X	X		12	0.11
	X		X		X	3	0.03
	X		X			1	0.01
Pakistan	X	X	X	X	X	3197	95.35
	X	X	X	X		129	3.85
	X	X	X		X	7	0.21
	X	X	X			2	0.06
	X		X	X	X	10	0.30
	X		X	X		8	0.24
Bangladesh	X	X	X	X	X	1082	97.92
	X	X	X	X		15	1.36
	X	X	X		X	2	0.18
	X		X	X	X	2	0.18
	X		X	X		4	0.36
African	X	X	X	X	X	880	15.28
	X	X	X	X		3176	55.13
	X	X	X		X	4	0.07
	X	X	X			4	0.07
	X		X	X	X	50	0.87
	X		X	X		1289	22.37
	X		X			358	6.21
Caribbean	X	X	X	X	X	8399	43.72
	X	X	X	X		4341	22.59

(continued)

Ethnicity	A	C	B	DRB1	DQB1	Count	Fraction (%)
	X	X	X		X	29	0.15
	X	X	X			9	0.05
	X		X	X	X	477	2.48
	X		X	X		3863	20.11
	X		X		X	1	0.01
	X		X			2094	10.90
Jewish	X	X	X	X	X	3517	35.23
	X	X	X	X		1640	16.43
	X	X	X		X	6	0.06
	X	X	X			3	0.03
	X		X	X	X	320	3.21
	X		X	X		3957	39.63
	X		X		X	3	0.03
	X		X			538	5.39
East Asian	X	X	X	X	X	2889	67.47
	X	X	X	X		870	20.32
	X	X	X		X	7	0.16
	X	X	X			5	0.12
	X		X	X	X	39	0.91
	X		X	X		394	9.20
	X		X			78	1.82
Middle Eastern	X	X	X	X	X	651	44.93
	X	X	X	X		594	40.99
	X		X	X	X	2	0.14
	X		X	X		192	13.25
	X		X			10	0.69
