# Supplementary material for: The HLA diversity of the Anthony Nolan register
Source: HLA. 2020 Nov 16;97(1):15–29. doi: 10.1111/tan.14127 (PMC7756289; doi:10.1111/tan.14127)
Supplement: Supplementary file 3 — Table S3 Supporting Information [file TAN-97-15-s003.pdf]

S 3: Nei's pairwise genetic distance matrix between subpopulations of the AN register

	African	Asian	Bangladesh	BINWE	Caribbean	India	Jewish	Middle Eastern	East Asian	Pakistan
African	0.00	3.63	4.58	2.31	0.23	3.76	3.40	2.65	3.78	3.76
Asian	3.63	0.00	0.43	2.08	2.47	0.02	2.11	1.12	1.01	0.10
Bangladesh	4.58	0.43	0.00	2.91	2.84	0.54	3.05	2.35	1.07	0.87
BINWE	2.31	2.08	2.91	0.00	1.24	2.25	1.28	1.08	2.77	2.30
Caribbean	0.23	2.47	2.84	1.24	0.00	2.56	2.41	1.91	2.96	2.70
India	3.76	0.02	0.54	2.25	2.56	0.00	2.17	1.15	1.14	0.11
Jewish	3.40	2.11	3.05	1.28	2.41	2.17	0.00	0.99	2.97	2.19
Middle Eastern	2.65	1.12	2.35	1.08	1.91	1.15	0.99	0.00	2.06	1.09
East Asian	3.78	1.01	1.07	2.77	2.96	1.14	2.97	2.06	0.00	1.28
Pakistan	3.76	0.10	0.87	2.30	2.70	0.11	2.19	1.09	1.28	0.00
