# Supplementary material for: The HLA diversity of the Anthony Nolan register
Source: HLA. 2020 Nov 16;97(1):15–29. doi: 10.1111/tan.14127 (PMC7756289; doi:10.1111/tan.14127)
Supplement: Supplementary file 4 — Supporting Information [file TAN-97-15-s004.zip › Supplementary README.PDF]

The HLA diversity of the Anthony Nolan register: Supplementary information

Frequency files

- **freqs_african_results.xlsx**: Allele, haplotype, and phenotype frequencies for African population.
- **freqs_asian_results.xlsx**: Allele, haplotype, and phenotype frequencies for Asian population.
- **freqs_bangladesh_results.xlsx**: Allele, haplotype, and phenotype frequencies for Bangladesh population.
- **freqs_binwe_results.xlsx**: Allele, haplotype, and phenotype frequencies for BINWE population.
- **freqs_caribbean_results.xlsx**: Allele, haplotype, and phenotype frequencies for African Caribbean population.
- **freqs_eastasian_results.xlsx**: Allele, haplotype, and phenotype frequencies for East Asian population.
- **freqs_india_results.xlsx**: Allele, haplotype, and phenotype frequencies for Indian population.
- **freqs_jewish_results.xlsx**: Allele, haplotype, and phenotype frequencies for Jewish population.
- **freqs_middleeastern_results.xlsx**: Allele, haplotype, and phenotype frequencies for Middle Eastern population.
- **freqs_pakistan_results.xlsx**: Allele, haplotype, and phenotype frequencies for Pakistan population.

where each .xlsx workbook contains sheets:

- **Allele frequencies (HLA-A)**: Complete list of HLA-A allele frequencies.
- **Allele frequencies (HLA-C)**: Complete list of HLA-C allele frequencies.
- **Allele frequencies (HLA-B)**: Complete list of HLA-B allele frequencies.
- **Allele frequencies (HLA-DRB1)**: Complete list of HLA-DRB1 allele frequencies.
- **Allele frequencies (HLA-DQB1)**: Complete list of HLA-DQB1 allele frequencies.
- **Haplotype frequencies**: 5 locus haplotype (A~C~B~DRB1~DQB1) frequencies above 0.0001.
- **Phenotype frequencies**: 5 locus phenotype (A~C~B~DRB1~DQB1) frequencies above 0.0001.
